# Evaluation of Effect of Ti Addition to Zinc Bath on Kinetics of Growth of Alloy Layer Formed in Process of Hot-Dip Galvanisation on Steel Substrate

**DOI:** 10.3390/ma16134773

**Published:** 2023-07-01

**Authors:** Karolina Bracka-Kęsek, Andrzej Szczęsny, Edward Guzik, Dariusz Kopyciński

**Affiliations:** Faculty of Foundry Engineering, AGH University, al. Adama Mickiewicza 30, 30-059 Krakow, Poland

**Keywords:** hot-dip galvanising, HDG with addition titanium, intermetallic phase Zn-Fe-Ti

## Abstract

Faced with the raw material crisis in Zn resources, researchers are facing the challenge of developing technology for producing zinc coatings that are thinner than those that have been produced to date. This would make it possible to reduce Zn consumption in the hot-dip galvanisation process. The study included an experiment that involved dip galvanising steel samples in baths of different Ti concentrations; this process was carried out at 450 °C and 550 °C. The use of this additive made it possible to reduce the growth of the alloy layer in the obtained zinc coatings. Using an optical microscope, observations were made of the microstructures of the resulting coatings, which made it possible to determine the thickness of the alloy layer in the coating. Thanks to the use of scanning electron microscopy with EDS analysis, however, it was possible to plot the chemical composition of the studied coatings and accurately observe the morphology of the formed phases. An intermetallic Zn-Fe-Ti phase was observed in the coatings formed in a Ti-added bath, which can affect the growth inhibition of the alloy layer in the zinc coating.

## 1. Introduction

The hot-dip galvanising method (HDG) is the most popular method for protecting steel products from corrosion. The susceptibility of steel products to corrosion forces manufacturers to use various methods of corrosion protection. These include all kinds of immersion metallisation methods and traditional painting. Often a combination of these methods is used. Galvanising, due to its uncomplicated manufacturing technology, was quickly popularised and began to be used on a large scale. Zinc consumption currently reaches 14 million tons per year, of which as much as 50 wt.% is used for dip galvanising [1]. Zinc deposits are already heavily depleted; hence, the need to find a dip-galvanising method that would reduce its consumption in the process. Zinc bath additives are being developed by researchers around the world that use different mechanisms of elemental action on the kinetics of zinc-coating formation; these include elements such as tin, magnesium, nickel, aluminium, bismuth, and lead. These additives have various effects. They inhibit the growth of intermetallic phases, modify the resulting phases, or alter corrosion properties, for example, by modifying the eta phase. Zinc bath additives affect not only the resulting coatings but also the conduct of the process by changing the physical properties of the liquid metal [2,3,4,5,6,7,8,9].

Tin is an element that improves the viscosity of metal and reduces the surface tension of such a bath; it has a beneficial effect on Zn consumption in the process, and the resulting coatings are characterised by a more aesthetic, smoother surface finish [6].

By using the addition of magnesium, the effect of grain fragmentation in the η-phase layer was achieved. In addition, magnesium, together with aluminium, causes the formation of dendritic grains, which improve the corrosion resistance of the obtained coatings [10].

Nickel is also considered an element with beneficial effects on the immersion galvanisation process. The addition of this element to a zinc bath at an amount of 0.06 wt.% causes a change in the kinetics of the phase formation and its morphology. In addition, the preservation of the Sandelin effect and the higher corrosion resistance of zinc coatings with Ni additives have been observed [11].

Zinc-aluminium coatings have been studied across the spectrum of Al contents in Zn (up to pure aluminium coatings). Reinforcing bars that have been galvanised in zinc baths with 10 wt.% Al added have shown better corrosion resistance than those that have been galvanised in pure zinc [12]. The addition of 0.8 wt.% Al to a zinc drip causes the formation of an inhibitory nano-layer on the substrate surface; this delays the build-up of the zinc coating [13].

Bismuth was found to have a beneficial effect on the casting properties of such baths by reducing the surface tension of the drip. As a result, this has been shown to reduce Zn consumption in the process by reducing the thickness of the η phase layer [6,10].

It is also worth considering the synergetic effect of alloying additives to zinc baths during the HDG process [10]; however, there are also negative effects of using these additives. A decrease in corrosion resistance, or susceptibility of coatings to white rust, can be observed. Today’s industry uses commercial alloys that contain the above-mentioned additives. Manufacturers are developing optimal chemical compositions for these baths, so there is no need to prepare baths in galvanising plants. This guarantees the high reproducibility of the immersion galvanisation process and the high quality of the coating.

There are no studies on the effect of adding Ti alone to a zinc bath on steel substrates of this grade, so such a topic has been addressed. Other additives may distort the picture of the effect of Ti not only on the microstructure of coatings but also on corrosion resistance. However, more factors need to be taken into account than the mere chemical composition of the zinc bath. The influence of the type of substrate on the formation of the zinc coating was found. It was also shown that the rougher the substrate that is subjected to dip metallisation, the thicker the coating is [14,15].

This paper describes an experiment that was conducted that showed that the addition of Ti to zinc baths causes the formation of a Ti-rich intermetallic phase layer. This phase can result in blocking the growth of Fe-Zn intermetallic phases on the steel substrate. In addition, there are potentially more advantages to such coatings, such as greater corrosion resistance, a more aesthetically pleasing surface finish, and better mechanical properties.

## 2. Materials and Methods

**Figure 1:**
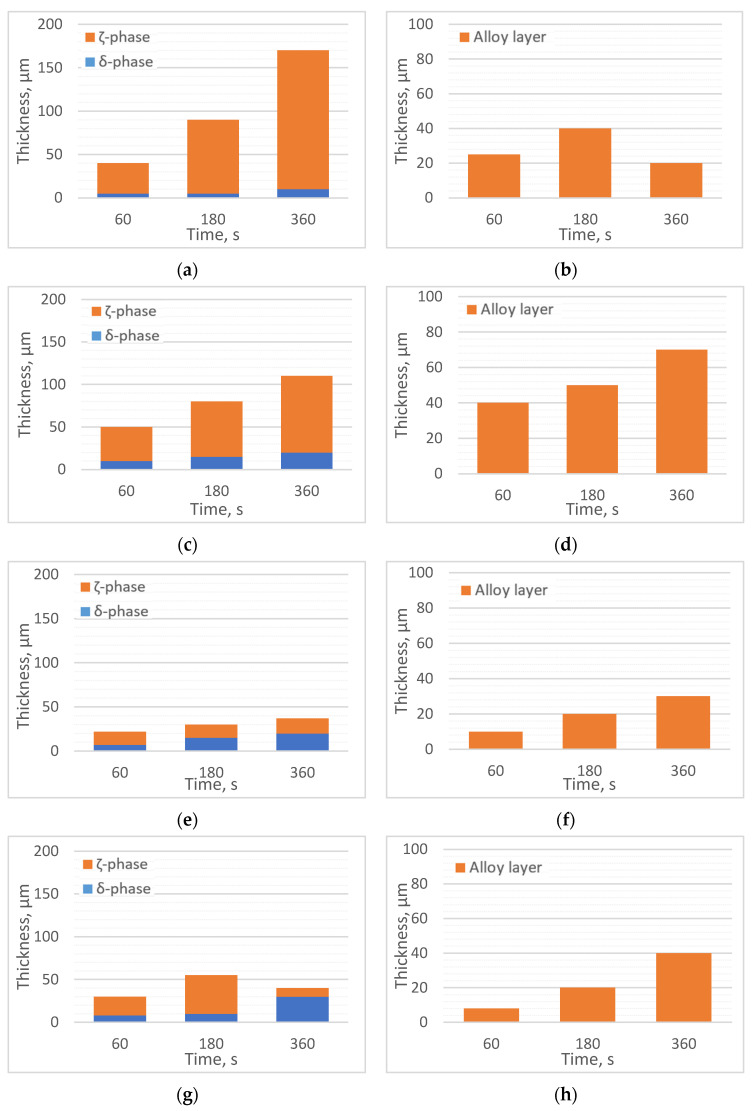

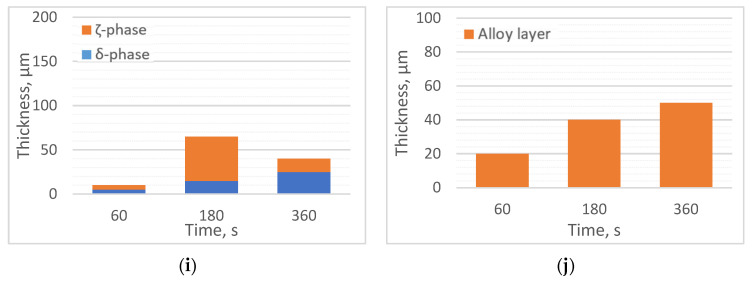
Thicknesses of alloy layers: (**a**) 450 °C, Bath 0; (**b**) 550 °C, Bath 0; (**c**) 450 °C, Bath A; (**d**) 550 °C, Bath A; (**e**) 450 °C, Bath B; (**f**) 550 °C, Bath B; (**g**) 450 °C, Bath C; (**h**) 550 °C, Bath C; (**i**) 450 °C, Bath D; (**j**) 550 °C, Bath D.

The purpose of the study was to investigate the effect of different Ti contents in the zinc bath on coating formation. In addition, only a Ti additive was used, without using other elements as alloying additives to the bath. Zinc baths with Ti additions were considered in the experiment. Bath galvanisation without Ti addition was also carried out to obtain reference results. First, the Fe-Ti equilibrium system in Figure 1 was analysed, which was a system with limited solubility of titanium in zinc. The melting point of pure titanium is 1668 °C, so its solubility in zinc within the temperature range of the zinc-plating process was very low. We could observe the existence of a number of peritectics starting at a temperature of 418.6 °C. The existence of a Zn_16_Ti eutectic at 418.6 °C could also be observed. The eutectic point on the diagram occurred for a 0.2 wt.% Ti content. In view of the above analysis, we could observe the precipitation of these Zn-Ti phases in a zinc coating growing on a steel substrate in a bath with small additions of Ti. It was possible that these phases were already formed to some extent in the zinc bath.

The experiment assumed the addition of titanium to the baths at different concentrations. The reference melt—with no Ti added—gave a suitable background for analysing the resulting coatings. The baths were prepared from pure components—electrolytic zinc ZnII (99.99 wt.% Zn) and recycled titanium sheets. The assumed composition of the prepared zinc baths is shown in Table 1.

The temperature of the immersion galvanising process is a determining factor in the order of the formation of the intermetallic phases on the steel substrate; in addition, the solubility factor of Ti in Zn must also be taken into account when Ti is added to a bath. Low-temperature galvanisation is characterised by the separation of the ζ-phase, followed by the δ-phase; this is due to the diffusion of the atoms. In the high-temperature process, there is the formation of only the δ phase [16]. When selecting the temperature of the process for the corresponding structure, it is important to remember that it affects the strength of the galvanised products [17].

Due to the chemical composition that is shown in Table 2, we can classify the steel substrate to be above the Sandelin range (where no extensive coating growth should occur due to the silicon content of the substrate). Over-Sandelin steel was used, so the resulting zinc coatings should not have been excessive [18]. Despite this, significant changes were observed in the microstructure of the resulting coatings.

The outer layer of the coating—the eta phase—usually accounts for about 30% of the total coating thickness. Its thickness can be adjusted (although this is difficult) when pulling the sample from the bath or by using alloy additives that reduce the surface tension of the liquid metal. The thickness of the alloy layer defines the time the sample is immersed in the bath. The time to form a continuous alloy layer over the entire surface of the product can carry the danger of growing too thick, thus increasing Zn consumption in the process. Our hypothesis is as follows: the Ti content causes a decrease in the diffusion between Fe and Zn atoms, resulting in a decrease in the rate of alloy layer growth.

Performing the process in the following manner allowed us to properly prepare the substrate, and the cooling in water allowed us to freeze the crystallisation at the desired stage. In this way, we did not have a disturbed picture of the formation of the coating through the further phase transformations or the oxidation of the layer. The oxidation of the resulting coating (which contained Ti) could be properly controlled, and the oxidised layer should be of the appropriate colour depending on the thickness of the layer. Temperature is the parameter that determines the rate of surface oxidation. The higher the temperature, the thicker the oxide layer formed. The thickness of the oxide layer clearly determines the colour obtained on the surface [19,20,21].

A study was developed that analysed immersion galvanisation in a 0.15 wt.% bath. The difference in the cooling of the samples is important. Cooling in the air not only allows for the free oxidation of the surface but also the further transformation in the solid phase of the coating and the diffusion of elements [20]. Zinc coatings have also been obtained in baths with 1 wt.% Ti, but it appears that such a high content promotes the growth of large Zn-Fe-Ti intermetallic phase precipitates (this also has an adverse effect on the mechanical properties and the formation of stresses within the coating [22]). In addition, such a Ti content in a bath also leads to the intense oxidation of the metal mirror [22]. An analysis of the literature also pointed to a paper that included a 0.5 wt.% addition. Also observed was a higher reactivity of the bath than in the zinc bath without additives [23].

Our machined steel substrate was properly prepared before immersion in the bath:

- Samples were immobilised on steel wires;
- Surface cleaned and degreased in ethanol (98 wt.%);
- Etched with HCl for 10 min;
- Fluxed in 35% aqueous solution of ZnCl_2_/NH_4_Cl salt mixture at 70 °C for 5 min;
- Dried of flux in a laboratory dryer for a minimum of 10 min;
- Immersion time and extraction time ware 2 s each;
- Dipped in a zinc bath for the appropriate time (60, 180, or 360 s);
- Cooled in water.

The baths were prepared in a ceramic crucible that was heated by resistance. Such crucible material was used because of the intensified reactivity of the zinc bath with steel containers. The stove is equipped with an automatic temperature controller. We assume that the temperature of conducting the process varied ±10 °C from the set temperature.

## 3. Results

### 3.1. Thickness of Obtained Zinc Coatings

The galvanised specimens were cut to reveal the cross-sections of the zinc coatings (encapsulated in resin); then, the metallographic samples were prepared using a laboratory grinder. After grinding and polishing, the samples were etched with Vilell’s reagent to reveal the interfacial boundaries for 1 s. The specimens were rinsed in ethanol and dried.

From the microstructural images, the approximate thicknesses of the alloy layers of the obtained coatings were measured (up to 5 μm). The results of the coating-thickness measurements are shown in Figure 1.

#### Scanning Microscope Examination with Chemical Composition Analysis

**Figure 2:**
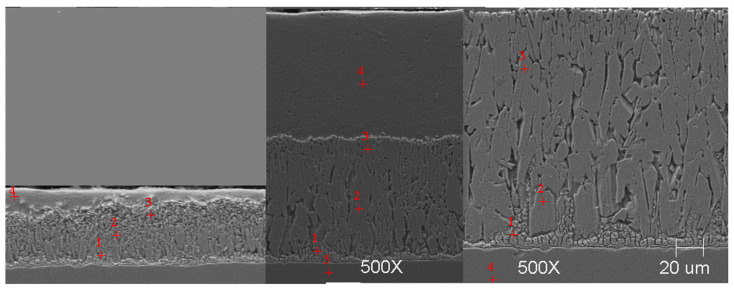
Microstructure of obtained coating in Bath 0 at 450 °C (from left—60, 180, and 360 s).

**Figure 3:**
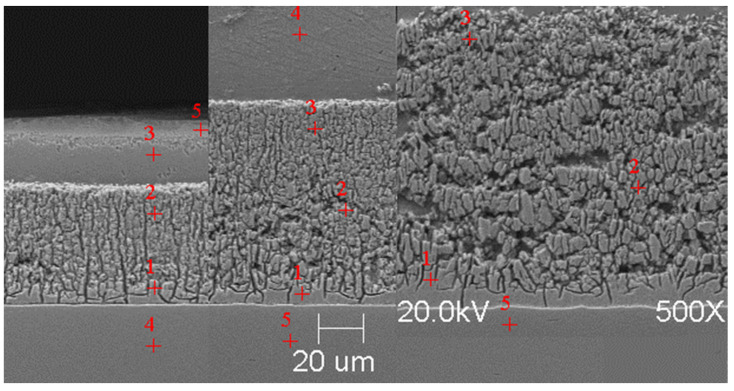
Microstructure of obtained coating in Bath A at 450 °C (from left—60, 180, and 360 s).

**Figure 4:**
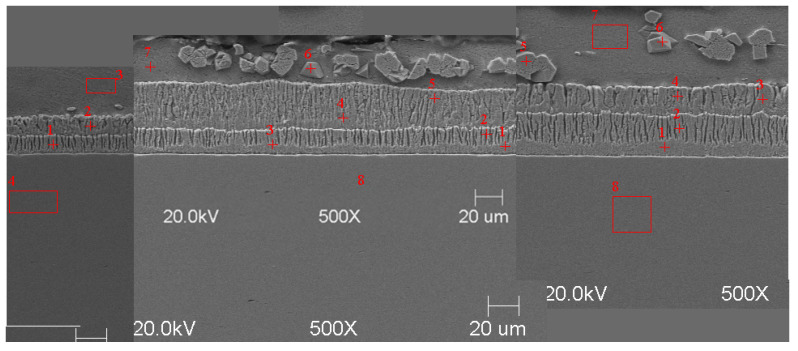
Microstructure of obtained coating in Bath B at 450 °C (from left—60, 180, and 360 s).

**Figure 5:**
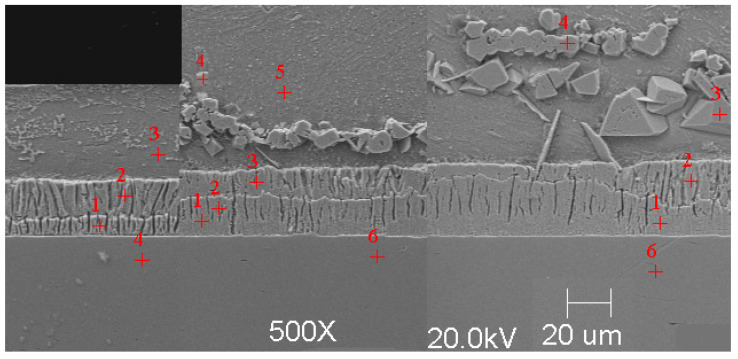
Microstructure of obtained coating in Bath C at 450 °C (from left—60, 180, and 360 s).

**Figure 6:**
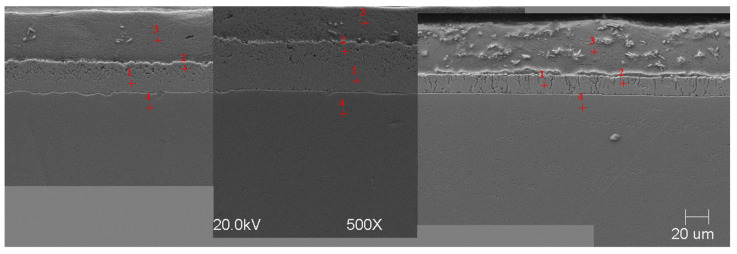
Microstructure of obtained coating in Bath 0 at 550 °C (from left—60, 180, and 360 s).

**Figure 7:**
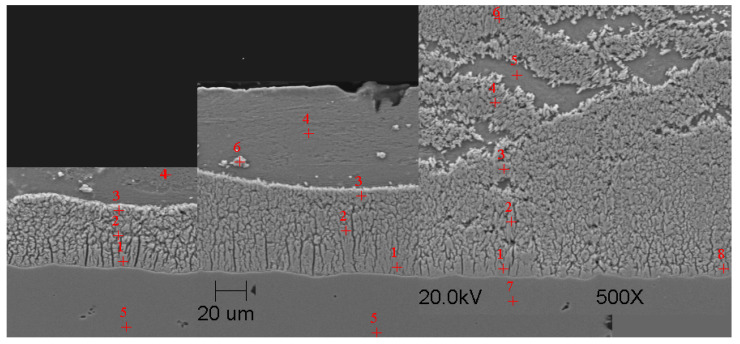
Microstructure of obtained coating in Bath A at 550 °C (from left—60, 180, and 360 s).

**Figure 8:**
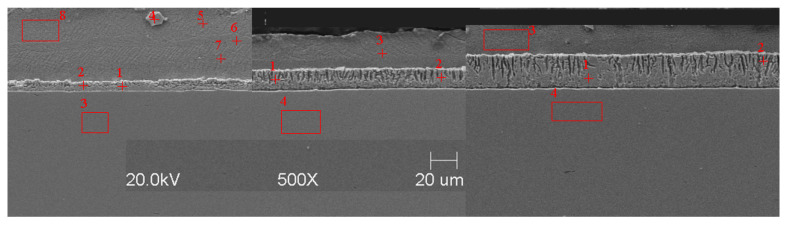
Microstructure of obtained coating in Bath B at 550 °C (from left—60, 180, and 360 s).

**Figure 9:**
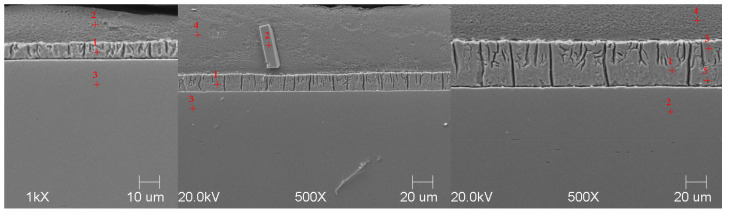
Microstructure of obtained coating in Bath C at 550 °C (from left—60, 180, and 360 s).

**Figure 10:**
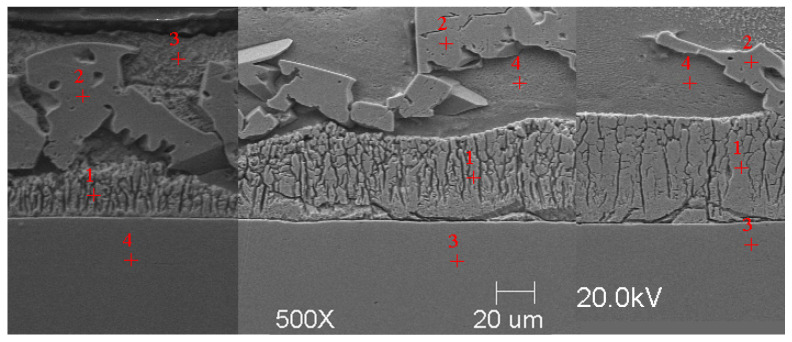
Microstructure of obtained coating in Bath D at 550 °C (from left—60, 180, and 360 s).

Examining the metallographic specimens with a JEOL 500LV scanning microscope (JEOL Ltd., Tokyo, Japan) allowed the microstructures of the coatings to be more accurately captured, the morphologies of the resulting phases to be analysed, and the chemical compositions to be determined by using an X-ray microanalysis attachment. The samples were previously etched more deeply with Vilell’s reagent—up to a minute; they were then subjected to ultrasonic washing in ethyl alcohol and dried in a laboratory dryer. The images of the microstructures that were taken by electron microscopy are shown in Figure 2, Figure 3, Figure 4, Figure 5, Figure 6, Figure 7, Figure 8, Figure 9 and Figure 10. Spot chemical composition analysis is provided in Table 3, Table 4, Table 5, Table 6, Table 7, Table 8, Table 9, Table 10 and Table 11.

**Figure 11:**
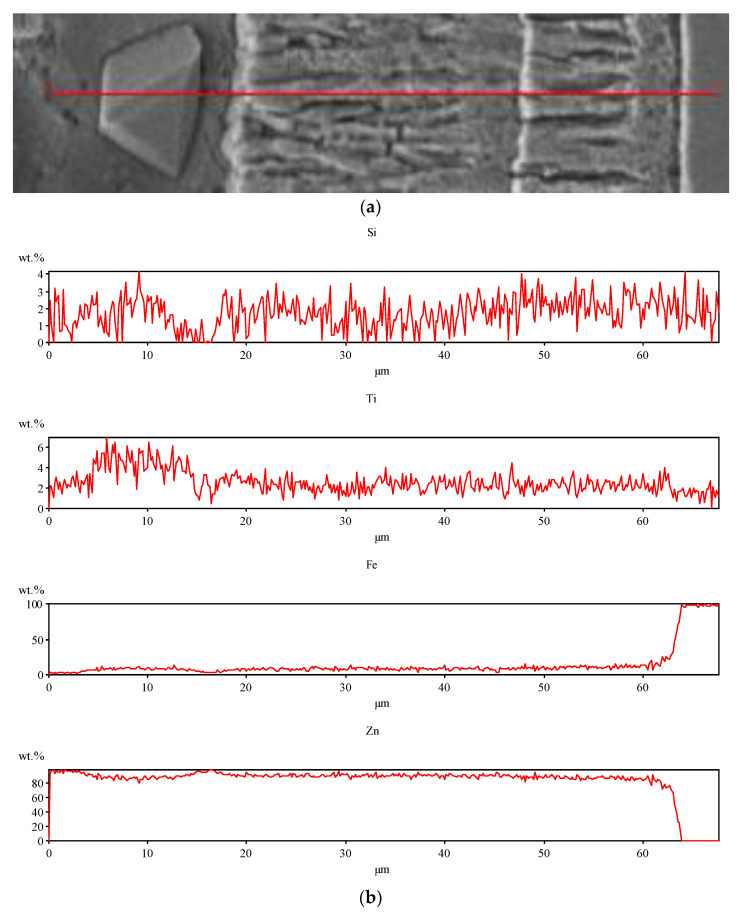
Results of analysis with LineScan tool—Bath B, the temperature of 450 °C, and immersion time of 180 s: (**a**) study area; (**b**) distribution of chemical composition in the study area.

Spot and area analysis of the chemical composition allowed us to determine the types of phases that were present in the coatings grown in the Ti-added drips. The observed microstructures were characterised by a layered structure. On the samples, we can observe growing δ- and ζ-phases from the substrate from the process that was carried out at 450 °C. The samples that were galvanised at a temperature of 550 °C were characterised by the growth of a uniform alloy layer (likely consisting of a delta phase). This phase δ from the ground is compact, while further from the ground, it is palisaded. In Figure 11, we can observe cracks inside the coating along the substrate. They are probably formed due to the variable thermal expansion of the phases formed in the coating with the addition of Ti. Table 12 shows the results of the spot analyses of the chemical compositions for the observed Zn-Fe-Ti phase precipitates.

A spot analysis was also performed along a line using the LineScan tool. Figure 11 shows an example of the results of the analysis that was performed for a coating that was formed in Bath B at 450 °C for 180 s. 

This kind of tool allows you to visualise the elemental content following a line drawn on the figure. An even proportion of Ti in the alloy layer (delta and dzeta phases) and a higher proportion of Ti in the separation were observed. In addition, a negligible share of Si in the eta phase was noted, while in the alloy layer, it remains constant regardless of the phase.

## 4. Discussion

In each of the coatings, we can observe the growth of an alloy layer on the surface of the steel substrate (which usually includes δ and ζ phases). In addition, those coatings that were formed in Baths B, C, and D at 450 °C featured the occurrence of an unknown Fe-Ti-Zn phase. At 550 °C, separations of this type were observed in the coatings that were formed in Bath D. As the immersion time increased, the separations of this phase were greater. Analyses of the chemical compositions of these precipitates showed Ti contents from about 2 to as much as 4 wt.%. A small Ti content was also observed in the δ and ζ phases that were formed in the alloy layer. 

The alloy layers of those coatings that were obtained in the baths with Ti additives were thinner than those that were dipped in pure Zn (in those processes that were carried out at both 450°C and 550 °C). The smallest alloy layer thickness was obtained at 550 °C in Bath B. At 450 °C, the coating that was obtained in Bath B also had the smallest thickness.

As the immersion times of the samples in the bath increased, the thicknesses of the resulting coatings increased. The one exception was the test that was carried out in the melt without Ti at 550 °C (where we can infer the greater diffusion of atoms from the substrate to the bath).

The structures of the resulting coatings were mostly layered. Only in Smelting A could the formation of a thicker layer be observed (which was a mixture of eta and zeta phases). Those coatings that were obtained in the B/C/D baths were compact and continuous.

The processes that were carried out at higher temperatures resulted in the formation of overall thinner alloy layers of coatings—bypassing the ζ-phase. In these cases, however, there was a danger of creating more waste in the form of hard zinc. For this reason, it is worth considering the shortest possible immersion time in the bath in a high-temperature process.

A Linescan analysis determined the Ti content on the cross-section of the coating. A higher content could be observed in the separations above the alloy layer. In the alloy layer, the Ti content remained constant; however, it is worth noting that this content was much higher than that which was declared in the bath.

## 5. Conclusions

The thesis confirmed that the addition of Ti in the galvanising process results in the growth of thinner coatings. The thesis was confirmed—already for small Ti contents in the zinc bath (as in the case of Bath B), there was a limitation of the alloy layer growth in the coating. However, in the case of Bath D, the Ti content resulted in the occurrence of large Zn-Fe-Ti phase precipitates. Accurate identification of this phase can be made difficult due to the large number of elements in the elemental cell as well as the complexity of the structure.

The future of dip galvanising belongs to zinc drip additives, which will reduce Zn consumption in the process and perhaps also have beneficial effects on the other properties of the resulting coatings (such as corrosion resistance and mechanical properties).

A subject of further research will be an attempt to accurately identify the observed Zn-Fe-Ti phase precipitates, as well as a series of corrosion tests.

## Figures and Tables

**Table 1:** Addition of titanium to zinc baths.

Bath	Addition of Titanium
0	0
A	0.01%
B	0.05%
C	0.1%
D	0.5%

**Table 2:** Chemical composition of steel.

Element	C	Mn	Si	P	S	Cr	Ni	Cu	Mo	Al	Ti	V
wt.%	0.16	0.5	0.18	0.026	0.013	0.07	0.11	0.31	0.02	0.002	0.001	0.002

**Table 3:** Results of chemical composition analysis in Bath 0 at 450 °C.

	60 s			180 s			360 s		
	Si	Fe	Zn	Si	Fe	Zn	Si	Fe	Zn
1	0.22	6.471	93.309	0.294	7.202	92.504	0.22	6.732	93.048
2	0.299	4.91	94.791	0.411	4.839	94.75	0.128	4.973	94.9
3	0.575	2.353	97.072	0.163	4.463	95.374	0.308	5.47	94.222
4	0.461	0.082	99.457	0.217	0.182	99.601	0.525	99.104	0.371
5				0.446	99.228	0.326			

**Table 4:** Results of chemical composition analysis in Bath A at 450 °C.

	60 s				180 s				360 s			
	Si	Ti	Fe	Zn	Si	Ti	Fe	Zn	Si	Ti	Fe	Zn
1	0.308	0.399	9.479	89.814	0.299	0.256	11.128	88.316	0.205	0.057	7.714	92.024
2	0.149	0.395	8.055	91.401	0.486	0.295	6.402	92.818	0.151	0.405	7.929	91.515
3	0.139	0.032	0.191	99.639	0.546	0.211	7.16	92.082	0.156	0.083	6.519	93.242
4	0.393	0.092	97.918	1.598	0.212	0.35	0.252	99.186	0.459	0.099	0.265	99.177
5	0.375	0.189	0.344	99.093	0.48	0	98.66	0.86	0.455	0.086	98.547	0.912

**Table 5:** Results of chemical composition analysis in Bath B at 450 °C.

	60 s				180 s				360 s			
	Si	Ti	Fe	Zn	Si	Ti	Fe	Zn	Si	Ti	Fe	Zn
1	0.662	0.277	9.576	89.485	0.453	0	9.642	89.905	0.729	0.649	10.042	88.58
2	0.354	0.117	5.629	93.9	0.65	0.118	7.79	91.443	0.287	0.495	6.957	92.262
3	0.132	0.283	0.1	99.485	0.406	0.261	8.75	90.583	0	0.391	5.562	94.046
4	0.346	0.102	99.423	0.13	0.249	0.128	5.369	94.255	0.433	0.23	4.27	95.067
5					0.177	0.677	5.028	94.117	0.119	3.449	6.141	90.29
6					0.253	1.849	3.683	94.214	0.187	2.837	6.375	90.6
7					0.142	0.273	0.11	99.475	0.208	0.449	0.539	98.804
8					0.532	0.238	99.23	0	0.55	0.025	98.442	0.984

**Table 6:** Results of chemical composition analysis in Bath C at 450 °C.

	60 s				180 s				360 s			
	Si	Ti	Fe	Zn	Si	Ti	Fe	Zn	Si	Ti	Fe	Zn
1	0.432	0.121	9.929	89.519	0.245	0.093	11.009	88.653	0.182	0.257	9.778	89.783
2	0.638	0.259	6.328	92.776	0.388	0.131	7.234	92.246	0.672	0.251	6.215	92.863
3	0.128	0.123	0.339	99.411	0.461	0.182	6.178	93.179	0	4.016	5.542	90.443
4	0.779	0.148	98.513	0.56	0.167	3.128	6.031	90.674	0.198	3.374	5.972	90.456
5					0.516	0.217	0.148	99.065	0.038	0.379	0.289	99.295
6					0.287	0.108	99.29	0.315	0.459	0.026	98.601	0.915

**Table 7:** Results of chemical composition analysis in Bath 0 at 550 °C.

	60 s			180 s			360 s		
	Si	Fe	Zn	Si	Fe	Zn	Si	Fe	Zn
1	0.194	7.805	92	0.354	7.823	91.823	0.357	8.156	91.486
2	0.389	6.491	93.119	0.401	6.962	92.636	0.098	7.161	92.741
3	0.322	0.31	99.365	0.391	0.424	99.184	0.295	0.053	99.653
4	0.484	98.935	0.582	0.24	98.706	1.054	0.573	98.849	0.578

**Table 8:** Results of chemical composition analysis in Bath A at 550 °C.

	60 s				180 s				360 s			
	Si	Ti	Fe	Zn	Si	Ti	Fe	Zn	Si	Ti	Fe	Zn
1	0.204	0.144	10.351	89.301	0.272	0.15	9.614	89.965	0.121	0.035	7.677	92.168
2	0.179	0.296	7.006	92.519	0.087	0.034	6.899	92.98	0.092	0.091	5.164	94.653
3	0.144	0.424	6.244	93.188	0.153	0.373	5.598	93.871	0.172	0.084	5.4	94.344
4	0.1	0.058	0.161	99.681	0.089	0.1	0.111	99.699	0.179	0.03	5.153	94.637
5	1.088		98.912		1.25		98.75		0.135	0.022	0.228	99.615
6									0.037	0.213	4.421	95.329
7									1.212	0.105	98.192	0.491
8									0.222	0.049	7.723	92.006

**Table 9:** Results of chemical composition analysis in Bath B at 550 °C.

	60 s				180 s				360 s			
	Si	Ti	Fe	Zn	Si	Ti	Fe	Zn	Si	Ti	Fe	Zn
1	0.668	0.084	11.577	87.671	0.443	0.486	9.166	89.904	0.281	0.43	8.966	90.324
2	0.47	0.536	9.031	89.963	0.34	0.135	10.14	89.385	0.189	0.264	6.872	92.676
3	0.75	0.072	98.987	0.19	0.345	0.061	0.6	98.994	0	0.634	0.391	98.974
4	0.238	0.246	6.699	92.817	0.393	0.102	98.812	0.693	0.392	0.361	98.437	0.81
5	0.618	0.245	0.479	98.658								
6	0.111	0.537	0.371	98.981								
7	0.224	0.394	0.974	98.408								
8	0.337	0.139	0.399	99.124								

**Table 10:** Results of chemical composition analysis in Bath C at 550 °C.

	60 s				180 s				360 s			
	Si	Ti	Fe	Zn	Si	Ti	Fe	Zn	Si	Ti	Fe	Zn
1	0.264	0.453	11.361	87.922	0.149	0.022	8.022	91.807	0.284	0.143	9.705	89.867
2	0.314	0.229	0.881	98.577	0.391	3.352	6.582	89.676	0.367	0.127	98.739	0.766
3	0.149	0.135	99.379	0.337	0.247	0.119	98.878	0.756	0.432	0.138	7.107	92.323
4					0.121	0.143	0.348	99.387	0.373	0.184	0.801	98.642
5									0.302	0.277	11.755	87.666

**Table 11:** Results of chemical composition analysis in Bath D at 550 °C.

	60 s				180 s				360 s			
	Si	Ti	Fe	Zn	Si	Ti	Fe	Zn	Si	Ti	Fe	Zn
1	0.346	0.229	11.409	88.016	0.399	0.253	8.915	90.433	0.646	0.19	8.632	90.532
2	0.169	3.823	6.191	89.818	0.47	3.9	6.635	88.996	0.73	3.516	6.014	89.74
3	0.171	0.909	0.664	98.256	0.32	0	98.989	0.691	0.743	0.04	98.357	0.86
4	0.534	0.114	99.02	0.331	0.058	0.335	0.456	99.152	0.614	0.352	0.453	98.581

**Table 12:** Spot analyses of chemical compositions of observed Zn-Fe-Ti phase.

Measuring Point	Si	Ti	Fe	Zn
Figure 4, time 180 s, Point 6	0.253	1.849	3.683	94.214
Figure 4, time 360 s, Point 6	0.187	2.837	6.375	90.6
Figure 5, time 180 s, Point 4	0.167	3.128	6.031	90.674
Figure 5, time 360 s, Point 3	0	4.016	5.542	90.443
Figure 9, time 180 s, Point 2	0.391	3.352	6.582	89.676
Figure 10, time 60 s, Point 2	0.169	3.823	6.191	89.818
Figure 10, time 180 s, Point 2	0.47	3.9	6.635	88.996
Figure 10, time 360 s, Point 2	0.73	3.516	6.014	89.74

## Data Availability

Not applicable.

## References

[1] https://natural-resources.canada.ca/our-natural-resources/minerals-mining/minerals-metals-facts/zinc-facts/20534.

[2] Avettand-Fènoël M.-N., Reumont G., Goodwin F., Perrot P., Foct J. (2022). Effect of tin added to the zinc bath on the formation and the microstructure of hot-dip galvanized coatings. Int. J. Mater. Res..

[3] Kania H., Liberski P. (2008). Struktura i kinetyka wzrostu zanurzeniowych powłok cynkowych otrzymanych w kąpielach z dodatkiem cyny. Inżynieria Mater..

[4] Di Cocco V. (2012). Sn and Ti influences on intermetallic phases damage in hot dip galvanizing. Frat. Ed Integrita Strutt..

[5] Riener C.K., Raab A.-E., Luckeneder G., Rosner M. (2017). Zinc-Magnesium-Aluminium (ZM)-HDG-Coated Steel Sheet for Structural Parts to Outer Panels.

[6] Henryk K., Saternus M. (2023). Benefits and Limitations of the Use of Pb, Sn and Bi Alloying Elements in Hot Dip Galvanizing Bath: A Review. J. Mater. Eng. Perform..

[7] Grandhi S., Raja V.S., Parida S. (2021). Effect of manganese addition on the appearance, morphology, and corrosion resistance of hot-dip galvanized zinc coating. Surf. Coat. Technol..

[8] Kania H. (2017). Odporność korozyjna powłok otrzymanych w kąpieli ZnAl z dodatkiem Mg. Przemysł Chem..

[9] Kania H., Liberski P., Kwiatkowski L. (2010). Wpływ dodatku niklu w kąpieli do cynkowania na odporność korozyjną powłok zanurzeniowych. Ochr. Przed Korozją.

[10] Kheirifard R., Ahmadi N.P., Aghaie E., Khezrloo A., Tayebi M., Behnamian Y. (2022). Evaluation of the Corrosion Resistance of Hot-Dip Galvanized Magnesium and Aluminum Alloy Coating Using the Taguchi Method. J. Mater. Eng. Perform..

[11] Pistofidis N., Vourlias G., Konidaris S., Pavlidou E., Stergioudis G. (2007). The combined effect of nickel and bismuth on the structure of hot-dip zinc coatings. Mater. Lett..

[12] Al-Negheimish A., Hussain R.R., Alhozaimy A., Singh D.D.N. (2021). Corrosion performance of hot-dip galvanized zinc-aluminum coated steel rebars in comparison to the conventional pure zinc coated rebars in concrete environment. Constr. Build. Mater..

[13] Min T., Gao Y., Huang X., Gong Z., Li K., Ma S. (2018). Effects of aluminum concentration on the formation of inhibition layer during hot-dip galvanizing. Int. J. Heat Mass Transf..

[14] Szczęsny A., Kopyciński D., Guzik E. (2017). Shaping optimal zinc coating on the surface of high-quality ductile iron casting. Part I—Moulding technologies vs. zinc coating. Arch. Metall. Mater..

[15] Kopyciński D., Guzik E., Szczęsny A. (2017). Shaping optimal zinc coating on the surface of high-quality ductile iron casting. Part II—Technological formula and value of diffusion coefficient. Arch. Metall. Mater..

[16] Bicao P., Jianhua W., Xuping S., Zhi L., Fucheng Y. (2008). Effects of zinc bath temperature on the coatings of hot-dip galvanizing. Surf. Coat. Technol..

[17] Šmak M., Kubíček J., Kala J., Podaný K., Vaněrek J. (2021). The Influence of Hot-Dip Galvanizing on the Mechanical Properties of High-Strength Steels. Materials.

[18] Kania H., Liberski P., Podolski P., Gierek A. (2006). Cynkowanie stali z różną zawartością krzemu w kąpieli z dodatkiem niklu i bizmutu. Inżynieria Mater..

[19] Natali S., Volpe V., Zortea L., Burattini C., di Cocco V., Iacoviello F. (2015). Mechanical and Structural Characterization of Zn-Ti Colored Coatings. Procedia Eng..

[20] Takáts V., Hakl J., Csik A., Bereczki H.F., Lévai G., Godzsák M., Török T.I., Kaptay G., Vad K. (2017). Ti oxidation states in Zn(Ti) coating of hot-dip galvanized steels. Surf. Coat. Technol..

[21] Levai G., Godzsák M., Török T.I., Hakl J., Takáts V., Csik A., Vad K., Kaptay G. (2016). Designing the Color of Hot-Dip Galvanized Steel Sheet Through Destructive Light Interference Using a Zn-Ti Liquid Metallic Bath. Metall. Mater. Trans. A Phys. Metall. Mater. Sci..

[22] Bellini C., Carlino F., Natali S. (2019). Analysis of the Al and Ti additions influences on phases generation and damage in a hot dip galvanizing process. Procedia Struct. Integr..

[23] Bellini C., di Cocco V., Iacoviello F., Mocanu L.P. (2022). Impact of Copper, Tin and Titanium Addition on Bending-Induced Damage of Intermetallic Phases in Hot Dip Galvanizing. Metals.

